# Airport malaria: report of four cases in Tunisia

**DOI:** 10.1186/s12936-015-0566-x

**Published:** 2015-01-28

**Authors:** Emna Siala, Dhikrayet Gamara, Kalthoum Kallel, Jabeur Daaboub, Faiçal Zouiten, Sandrine Houzé, Aïda Bouratbine, Karim Aoun

**Affiliations:** Laboratoire de Parasitologie-Mycologie, Tunis, Tunisia; LR 11-IPT-06 «Parasitologie Médicale, Biotechnologies & Biomolécules», Institut Pasteur de Tunis, Tunis, Tunisia; Direction des Soins de Santé de Base, Tunis, Tunisia; Laboratoire de Parasitologie-Mycologie, Hôpital La Rabta, Tunis, Tunisia; Direction de l’Hygiène du Milieu et de Protection de l’Environnement, Tunis, Tunisia; Consultation privée d’infectiologie, Tunis, Tunisia; Malaria National Reference Centre, AP-HP, Bichât Hospital, Paris, France

**Keywords:** Airport malaria, *Plasmodium falciparum*, *Anopheles*, Tunisia

## Abstract

Four cases of airport malaria were notified for the first time in Tunisia during the summer of 2013. All patients were neighbours living within 2 km of Tunis International Airport. They had no history of travel to malarious countries, of blood transfusion or of intravenous drug use. Although malaria transmission had ceased in Tunisia since 1980, autochthonous infection by local *Anopheles* mosquitoes was initially considered. However, this diagnostic hypothesis was ruled out due to negative entomological survey and the absence of additional cases.

All cases were caused by *Plasmodium falciparum*. Clinical presentation was severe (important thrombocytopaenia and parasitaemia), because of relatively important delay in diagnosis (average of seven days). This indicates the need to consider malaria while examining airport employees or people living near international airports presenting with fever of unknown origin. It also stresses the need for effective spraying of aircrafts coming from malarious areas.

## Background

Malaria has been eliminated from Tunisia in 1979 [1]. Since then, no local cases have been notified. Recently, between 50 and 75 cases of imported malaria are registered yearly [2], mainly in travellers and migrants coming from malarious countries [3]. The present report refers to four local airport malaria cases in Tunisian citizens who had never been to endemic areas.

## Case reports

### Case 1

A 27-year-old man presented on 3 July 2013 with fever (40°C), myalgia and headache of two days standing for which he received paracetamol without improvement. The biological assessment showed WBC at 4,230/mm^3^, haemoglobin at 16 g/dL and thrombocytopaenia (85,000/mm^3^). Antibiotic therapy was prescribed. On 11 July, because of the persistence of fever, a second biological assessment was required and showed a thrombocytopaenia at 35,000/mm^3^. Blood smear performed to control thrombocytopaenia and check the presence of aggregated platelets revealed intra-erythrocytic trophozoites of *Plasmodium falciparum* with a parasitaemia at 1%. The patient was treated with Lumartem® (20 *mg* artemether/120 mg lumefantrine)(Cipla, India). He received a total of six doses (4 tablets per dose) over a period of three days, as recommended. The patient is a student, living within 2 km of Tunis international airport.

### Case 2

A 25-year-old friend and neighbour of case 1 presented on 6 July 2013 to the emergency department with fever, headache and myalgia accompanied by epistaxis. The diagnosis of angina was accepted and the patient was treated with amoxycillin. On the next day, the patient’s general condition deteriorated with vomiting, fever, and shivering. Laboratory investigations revealed leukocytopaenia at 2,300/mm^3^ with thrombocytopaenia (26,000/mm^3^) and abnormal liver function findings (ALAT 124UI/L, ASAT 129UI/L). Serological tests for influenza A and B virus were positive and Tamiflu was prescribed. On 11 July, malaria was suspected based on the diagnosis of case 1, and microscopic examination of blood smears confirmed the presence of *P. falciparum* with parasitaemia at 12%. The patient was treated for seven days with intravenous quinine dihydrochloride® (Rotexmedica, Germany) 8 mg/kg quinine base every eight hours after an initial loading dose of 17 mg/kg.

### Case 3

A 21-year-old man, living within 200 m from cases 1 and 2 was admitted to a private clinic with vomiting, shivering and muscular pain of one week’s standing. He had received paracetamol and metoclopramide (Primperan®). On 13 July, laboratory tests revealed thrombocytopaenia (26,000/mm^3^), leukocytes 4,600/mm^3^, C reactive protein at 163 mg/l, and abnormal liver function transaminase (ALAT 79UI/L, ASAT 50UI/L). In light of the two recent cases diagnosed in the same area, malaria was rapidly suspected and a microscopic examination of blood smears confirmed the presence of *P. falciparum* with a 0.5% parasitaemia level. The patient was treated with Lumartem®.

### Case 4

A 21-year-old man presented on 7 July with fever, myalgia, shivering and vomiting that had begun two days earlier and persisted despite treatment with paracetamol. Oral antibiotic therapy (amoxicillin/clavulanate) was prescribed. After an initial improvement, the patient's symptoms recurred with pain in the right hypochondrium. Consequently, he was admitted to hospital on 12 July, where physical examination revealed a slight spleen enlargement. Laboratory findings showed thrombocytopaenia at 38,000/mm^3^, leukocytopaenia at 3,800/mm^3^, C reactive protein at 220 mg/l and slightly elevated liver enzymes (ALAT 68UI/L, ASAT 62UI/L). Microscopic examination of blood smears was prescribed and revealed *P. falciparum* (parasitaemia = 0.01%). The patient was treated with Lumartem®.

History taking revealed that all cases lived in the same residential area within a radius of 2 km, south east of Tunis Carthage International Airport. They had no history of travel to malaria endemic areas or of blood transfusion. Repeated interviews eliminated also intravenous drug use. Three of the four patients were close friends. PCR testing confirmed *P. falciparum* in all cases. The genomic characterization of isolates was performed in the Malaria National Reference Centre Paris, France. It was performed on DNA extracted from blood samples by using the QIAamp DNA Blood kit (Qiagen, Germany) according to the manufacturer's recommendations. Five *P. falciparum* microsatellite loci (TAA 81, TAA 87, TAA 60, PKPK 1, and ARA 2) located on different chromosomes were genotyped using the method described by Musset *et al.* [4]. Genotyping showed similar plasmodial strains of *P. falciparum* in all extracts. Treatment led to rapid improvement in all cases and recovery was confirmed by negative control blood smears (Days 7, 14 and 28). As soon as the 1^st^ patient was diagnosed, active screening of febrile individuals living in the area (Berges du lac) was carried out for several months without detecting new cases of malaria.

### Mosquito investigation

An entomological survey was conducted in July 2013 during four days in the patients’ neighbourhood. Adult mosquitoes were caught using CDC light traps and larval stages were collected in available water collections. No *Anopheles* specimens were captured.

## Discussion

The four airport malaria cases diagnosed in Tunisia during the summer of 2013 concerned citizens born after the stop of *Plasmodium* parasite transmission. All patients had never travelled to malaria endemic areas and had no history of blood transfusion. The hypothesis of infection through intravenous drug use was also ruled out by careful history taking. Although eliminated from Tunisia since 1980, infection by local mosquito bites was initially considered as it had previously occurred in Algeria, Greece, Italy and United States [5-8]. In fact, a resurgence of the disease cannot be totally excluded because of the coexistence in Tunisia of imported malaria cases acting as a potential reservoir and local *Anopheles* representing potential vectors [9,10]. However, the entomological investigations carried out rapidly around the patient’s homes did not detect any anopheline insect. Moreover, the absence of additional cases in spite of active screening of the susceptible population ruled out the possibility of autochthonous transmission by local mosquitoes.

All four patients were geographically located in the same restricted district just 2 km distant from Tunis International Airport. Onset of symptoms occurred in a short period of less than one week. By ruling out infection by local mosquitoes, exchange of contaminated needles and blood transfusion, it was concluded that the transmission occurred through the bites of infected *Anopheles* mosquitoes transported by aircraft from a malaria endemic country. In fact, Tunis international airport is linked by regular flights to many West-African capitals where malaria transmission is common such as Abidjan, Bamako, Dakar, Nouakchott and Ouagadougou. Moreover, the local conditions, especially the temperatures in June 2013; corresponding to the supposed period of contamination (mean 23°7 C, range: 17°2 to 31°1 C), were auspicious for the survival and the activity of imported mosquito vectors [11].

Airport malaria is not an exceptional event. Between 1969 and 1999, 89 cases were reported in Europe mainly in France, Belgium, Italy, the Netherlands, Spain, Switzerland and UK [12-15]. Most infected patients lived near or worked in an international airport. Airport malaria occurred mainly in summer from June to September, when climatic conditions are favourable to mosquitoes [11]. The infected female mosquito leaving the aircraft, can survive long enough and can be dispersed under wind conditions from 7 to 15 km [16,17]. It could be transported further by vehicle and baggage [18,19].

Genotyping of the four *P. falciparum* isolates obtained from patients showed that a similar strain was involved. This finding could suggest several infections caused by the same mosquito taking an interrupted blood meal. Although rare, such event had been previously reported in 1999 with three malaria cases induced [20]. Bites by distinct anopheline females coming from the same malaria focus and infected by the same clone cannot be totally excluded.

Malaria is generally unsuspected by clinicians in patients from non-endemic countries who have never travelled to malarious areas. Hence, all four cases were diagnosed relatively late. Because of this delay (average of 7 days), *P. falciparum* aetiology and the naïve immune status of the patients, the observed clinical and biological presentations were severe (deep thrombocytopaenia, high parasitaemia). Therefore, malaria should be considered in case of high fever of unknown origin particularly in airport employees or people living near international airports.

Beyond the possibility of airport malaria cases, new direct flights between Tunisia and some sub-Saharan African countries increases the risk of invasion by highly competent vectors, as has happened with *Anopheles gambiae* in Brazil and Egypt [21,22]. This can lead to resurgence of malaria transmission [23]. Preventive recommendations, such as aircraft spraying should be followed strictly [24]. All potential larvae habitat in and around international airports and ports should also be eliminated or disinfected.

## Conclusion

The occurrence of airport malaria in Tunisia stresses the need for sensitization of clinicians for considering malaria in febrile individuals even when they have not travelled to an endemic area. Beside, this report highlights the importance of efficient disinfection of aircraft arriving from endemic areas.

## Consent

Written informed consent was obtained from the patient for publication of this Case report. A copy of the written consent is available for review by the Editor-in-Chief of this journal.
